# Passive Ultrasonic Irrigation Efficacy in the Vapor Lock Removal: Systematic Review and Meta-Analysis

**DOI:** 10.1155/2019/6765349

**Published:** 2019-03-12

**Authors:** Mario Dioguardi, Giovanni Di Gioia, Gaetano Illuzzi, Domenico Ciavarella, Enrica Laneve, Giuseppe Troiano, Lorenzo Lo Muzio

**Affiliations:** Department of Clinical and Experimental Medicine, University of Foggia, Via Rovelli 50, Foggia 71122, Italy

## Abstract

Passive Ultrasonic Irrigation (PUI) represents one of the most used systems to improve the endodontic irrigants activity. PUI acts increasing the reaction rate of NaOCl, with an increase of dentinal debris and smear layer removal. There is a stronger shear stress and a vapor lock reduction. Aim of this systematic review was to figure out the effects of the PUI on the vapor lock removal, during irrigation. Literature research has been carried out by two reviewers, consulting online databases such as PubMed, EBSCO, and Google Scholar, using keywords like Vapor Lock, Vapour Lock, and Vapor Lock Endodontic. The articles list has been screened based on titles and abstracts, applying eligibility and inclusion criteria. The three articles were eligible for quantitative and statistical analysis, by using RevManager Software Version 5.3. Results show statistical heterogeneity (P=0.08; I^2^ index=61%) in the vapor lock elimination between the use of PUI and PPI, with an overall Odds Ratio=0.08, CI=95% [0.03;0.25]. PUI resulted to be a useful technique to improve NaOCl activity for vapor lock removal, despite PPI alone using a needle.

## 1. Introduction

Aim of modern endodontics is to obtain the complete cleaning and disinfection of the root-canal system (RCS) in order to fulfill from the apical to the coronal sealing with a the 3D filling of the endodontium [1]. Many studies [2–4] show how an insufficient cleaning of the RCS can lead to an endodontic treatment failure. Bacteria organized in biofilms inside lateral canal scan lead to a lateral or apical periodontal lesion, even with the presence of an adequate apical sealing [5–7]; although, some studies prove the contrary [8, 9]. One of the main causes of secondary endodontic treatment failure is represented by the bacterium* Enterococcus faecalis* [10]. Many studies [11–14] show how this bacterium is able to withstand different NaOCl irrigation solutions. Factors that can contribute to an inadequate cleaning of the RCS can be of anatomical origin [15, 16], such as the presence of isthmi, anastomosis, and lateral canals, that may not be reached by the mechanical cleaning action of the majority of endodontic files [17–19]; other factors can be found in the procedural origin, like an incorrect shaping of the canal, leading to the formation of dentinal plugs and ledges and the absence of irrigation by NaOCl solution and smear layer removing irrigants [20]. Other causes that can negatively or positively influence cleaning procedures are of physical origin, such as reaction rate [21], shear stress [22], and vapor lock [23]. The increase of reaction rate improves the chemical properties of NaOCl, with a disinfecting action [24]. All methods increasing NaOCl action and its movement inside the RCSs can lead to the genesis of an oscillating/vibrating shear stress with a stronger impact on canal walls with a better removal of smear layer [25]. Vapor lock effect, main topic of this systematic review, is produced by disadvantageous events during the endodontic treatments. It is represented by the production of air or gas bubbles inside a close-ended system [26]; the formation of bubbles in the apical third of the canal leads to the impossibility of irrigant solutions to chemically reach those areas, with an increase of failures and relapses. Vapor lock is produced by both physical and chemical phenomena, like the release of CO_2_ by necrotic pulp tissues, being dissolved by NaOCl. Irrigation of RCSs is provided by the injection of disinfecting solution with the use of a syringe that should not be engaged to the canal walls: this technique is known as PPI. In PUI technique, irrigation is provided by an ultrasonic tip, which is introduced inside the canal, fulfilled with irrigant solution, and being activated in order to increase the cleaning and disinfecting action of NaOCl [27]. Endodontic practitioners are able to perform treatments faster than before, thanks to the introduction of nickel-titanium (NiTi) mechanical instruments files. The amount of time dedicated to chemical cleaning has been drastically reduced. It is now preferred to minimally shape the apex, with the use of 7-8% tapered and 0.20-0.25 mm of diameter instruments at the tip, in order to get a more predictable apical gutta-percha sealing to the detriment of irrigants action and possible increasing procedures [20], although other studies seem to prove the contrary [28, 29]. We aimed to revise the literature, following inclusion criteria: studies performed on that have artificial canals and extracted teeth, preventively shaped. We extracted data related to vapor lock formation and its eventual elimination after the adoption of techniques, improving NaOCl action, focusing more on the PUI method [30]. We will investigate if, using modern shaping techniques, there is a major incidence in vapor lock formation and if it is reduced using techniques improving irrigant solutions activity.

## 2. Materials and Methods

This systematic review has been performed according to the PRISMA protocol [31], including those studies, published between 1984 and 2017, comparing the use of PUI versus PPI, performed only on extracted teeth and evaluating the presence or absence of vapor lock (primary outcome).

### 2.1. Database Research

A direct research was performed using online databases, just as PUBMED, EBSCO, and Scholar Google, from December 2017 to February 2018. This has been performed looking for those articles that used teeth extracted because of pathological reasons and treated with the use of PUI, or as a treatment alternative, extracted teeth being treated with the use of PPI (standard therapy); the articles had also evaluated the presence of vapor lock after the use of the techniques mentioned above. The inclusion criteria applied are the following: English-written papers; studies with not less than 20 samples; studies made only using extracted teeth; studies using shaped teeth with, at least, an apical diameter of 20 mm and a taper of 6%.

### 2.2. Studies Selection

Studies selection has been executed in open by two independent reviewers. Conflicts have been resolved including a third reviewer in a joint session. Selection procedure has been done reading title and abstract only. All articles not showing relevance with the aims of the present review have been excluded. A qualitative analysis was performed for the included studies. Search strategy on PUBMED and EBSCO database has been performed using the following keywords: vapor lock; vapour lock. Scholar Google has been also consulted, typing in the search bar “vapor lock endodontic”, producing around 844 records: only one of them has been taken in exam. Later on, overlaps have been eliminated and articles not containing PUI have been excluded. Of these 5 articles, only 3 have been taken in consideration after the inclusion criteria application, in order to be used for quantitative analysis.

### 2.3. Risk of Bias Evaluation

Risk of bias has been evaluated using the Newcastle-Ottawa scale for case-control studies [32], due to a lack of a specific method for the risk of bias assessment for in vitro studies. Risk of bias between studies has been quantified, evaluating the presence of heterogeneity using Q-test and I^2^ index. Higgins index has been classified using the following values: <30% for low heterogeneity; from 30% to 70% for medium heterogeneity; >70% for high heterogeneity. Difference between groups was evaluated using the inverse of variance test. All calculations have been performed using Review Manager software, version 5.3 (Copenhagen, 153 Denmark, The Nordic Cochrane Centre, The Nordic Cochrane Collaboration, 2014). Results have been illustrated in forest plots. A p-value <0.05 has been considered significative for all tests used in this analysis.

## 3. Results and Discussion

**Figure 1:**
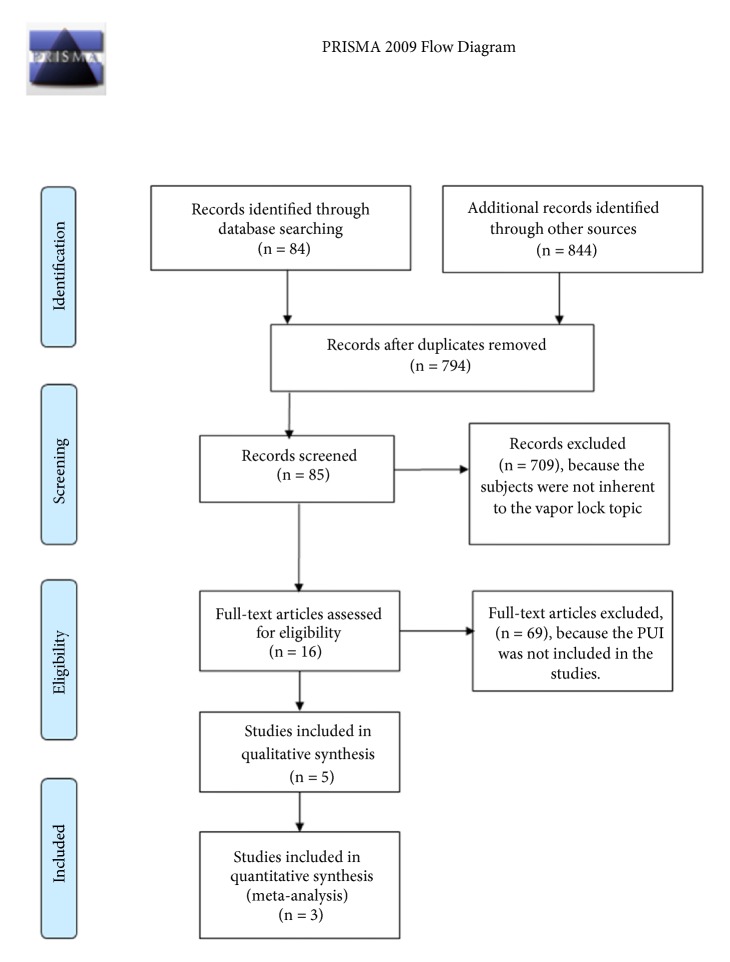
Flowchart describing the selection of studies used for the present systematic review, as described by the PRISMA guidelines.

Databases search produced 84 records, including 844 records from Scholar Google, for a total of 928 results; after the duplicates exclusion, the number of records reduced to 794. After the screening procedure, by reading both titles and abstracts, 709 of them have been excluded, obtaining a total of 85 records. Only 16 have been considered to be evaluable after excluding 69 of them. The reasons why these articles have been excluded are reported in the flow chart in [Figure 1 here]. These 16 articles have been read full-text, and only 5 of them were included in the qualitative synthesis. After the exclusion of 2 articles not containing PPI, the remaining 3 articles underwent quantitative analysis [Table 1 here].

**Figure 2:**
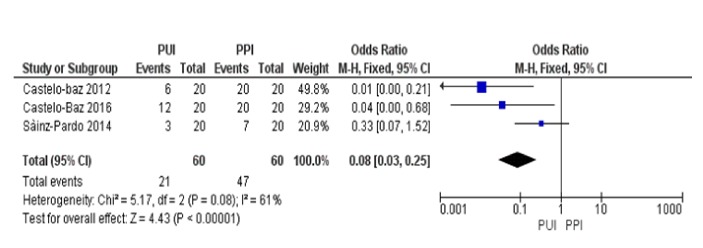
Results of risk of bias calculation between studies using Review Manager Software version 5.3, being illustrated with the help of forest plot graphics.

From each study, we extracted data related to the vapor lock presence (primary outcome) in every single sample, both for PUI and PPI methods. The risk of bias has been evaluated according to the Newcastle-Ottawa scale for case-control studies; a bias can be found between Sainz-Pardo study [33] and the other two by Castelo-Baz, where the one by Sainz-Pardo Estevez was structured using both open-ended and closed-ended RCSs, while those by Castelo-Baz used decalcified teeth, as reported by Robertson and Leeb [34]. According to the Newcastle-Ottawa scale for case-control, all the selected studies have an adequate case definition [Table 2 here]; studies by Castelo-Baz have an adequate representativeness of cases, while the one by Sainz-Pardo et al. are biased, due to the presence of both open-ended and closed-ended canals. Both selection and the definition of controls are not clear, while the comparability between cases and controls has been based on the vapor lock removal. For all studies, there is no description about the ascertainment of exposure, and they proved to be adequate both for method of ascertainment, related to cases and controls, and nonresponse rate. Results are illustrated in the forest plot graphic, and all the three studies are in favor of the PUI method. The study by Castelo-Baz [35], conducted using 60 extracted teeth, aimed to describe the presence of vapor lock using 3 methods: PUI, PPI, and Continuous Ultrasonic Irrigation (CUI) (the last method has not been investigated by the present review). Analysis has been performed using an irrigant contrast solution (composed of 5% NaOCl and 20% Chinese Ink); root-canal systems have been shaped using ProTaper Universal F3 files (Dentsply, Maillefer). 10 closed and open-system samples have been assigned to every single method to improve the irrigant efficacy. Results of this study show that, with the use of PUI, vapor lock was present in 6 samples, while with the use of PPI it was present in all samples, and with the use of CUI it was present in 4 samples; the study by Sainz-Pardo [33] has been conducted using 60 extracted teeth, half of them being open-ended RCSs and the other half being close-ended RCSs. 3 methods have been evaluated: PUI, PPI, and Sonic activation. 20 samples have been assigned to each of the methods, 10 being open-ended RCSs and 10 being close-ended RCSs. All samples have been shaped using Profile Rotatory Files, with an apical diameter and a taper of at least 0.30 mm and 6%, respectively; after delivering a contrast solution (composed of 5% NaOCl and 20% Chinese Ink) into the RCSs, all samples have been analyzed using X-ray exams. Results showed that, with the use of PUI, vapor lock was present in 3 close-ended RCSs samples and completely absent in open-ended RCSs samples; with the use of PPI, vapor lock was present in 7 close-ended RCSs samples and completely absent in open-ended RCSs samples; with the use of Sonic activation, vapor lock was present in 6 close-ended RCSs samples and completely absent in open-ended RCSs samples. The study by Castelo-Baz [36] was conducted using 60 extracted teeth, all being close-ended RCSs. 3 methods have been evaluated: PPI, PUI, and CUI. 20 samples have been assigned to each method. Each root canal was preflared using K-Flexofiles (Dentsply Maillefer, Ballaigues, Switzerland) with an apical diameter of at least 0.20mm and then shaped using this files sequence: GTX 20.04, 20.06, and 30.06 (Dentsply Maillefer); later on, after delivering a contrast solution (composed of 5% NaOCl and 20% Chinese Ink) into the RCSs, samples have been analyzed using X-ray exams). Results showed that, with the use of PUI, vapor lock was present in 12 samples; with the use of PPI, vapor lock was present in 20 samples; with the use of CUI, vapor lock was present in 2 samples. Carrying out data from all the 3 studies, the presence of vapor lock in 57/60 samples treated with the use of PPI has been proved. These results have been the subject of quantitative and statistical analysis using RevManager Software Version 5.3, and these have been illustrated using Forest Plots graphics: the analysis suggests a statistical heterogeneity (P=0.08; I^2^ index=61%) about the vapor lock elimination between the use of PUI and PPI, with an overall Odds Ratio=0.08, CI=95% [0.03;0.25] [Figure 2 here].

## 4. Main Text

Valuing the results of the qualitative analysis performed on those studies observing the vapor lock formation, it has to be noted that the genesis of this effect is strictly related to physical-chemical phenomena. An important factor in the vapor lock formation results being the production of gas bubbles, determined by organic tissues dissolution inside the canal caused by NaOCl action. This phenomenon is mostly present the apical third of a RCS and is mostly present in close-ended systems. There are many simple and cost-effective methods to improve the activity of irrigant solutions and their efficacy. One of them is the Manual Dynamic Activation (MDA) that consists in shaking the irrigant solution inside the canal, by moving a gutta-percha cone, adapted to the canal shape, with 2-3 mm amplitude movements inside and outside the canal. Otherwise, a manual tool such as a carrier, finger spreader, k-file, and even a syringe can be used (when using a syringe, one has to remember the presence of 2 different types of needles: those with the opening at the tip and those with the opening on their side; if used with side-opened needles [37], MDA contributes in increasing the vapor lock formation). Even though studies about vapor lock formation are few, it is clear how fundamental is improving the NaOCl activity using ultrasonic methods in order to reduce the treatment failure rate [38–40]. There are two methods of ultrasonic activation: the passive one, defined as Passive Ultrasonic Irrigation (PUI) that firstly delivers the irrigant solution into the RCS and then introduces the ultrasonic tip, without touching the canal walls; the second one, defined as (CUI) Continuous Ultrasonic Irrigation, where the activation of the irrigant solution is performed simultaneously with its delivery into the canal. PUI uses metallic inserts, capable of preserving the canal walls anatomy [30]. The vibration of the ultrasonic insert produces an acoustic stream that generates a shear stress, that is able to dislocate the debris inside instrumented RCSs. Inserts vibrate at a frequency of 25-30 kHz, since lower frequencies produce Sonic vibrations. Ultrasonic waves are able to propagate inside the irrigant solution and to create microcavitation (small voids) that implode, shaking the solution inside the canal and improving the removal of the smear layer as well as improving the penetration of the liquid into the apical third of the RCS. There is also the improvement of the reaction rate due to the irrigant solution higher temperature. Furthermore, PUI increases the removal of dentinal debris and smear layer, as seen in many studies [41–44]; on the other hand, it can lead more to the apical extrusion of NaOCl if used 2mm or shorter from the apical foramina or if stuck into canal walls. The CUI technique involves the outflow of the irrigant solution from a 25 G diameter needle that simultaneously vibrates at ultrasonic frequencies. This method has the advantage of increasing both the shear stress and the reaction rate, but there is an increased risk of extruding the irrigant solution beyond the apex. There are also Sonic systems to improve the irrigant efficacy inside RCSs, and so reducing the vapor lock formation: they cause the movement of the irrigating solutions that results in an improved cleaning of the RCSs, compared to the traditional irrigation with the simple syringe, but being inferior if compared to the ultrasonic method. This system increases the shear stress, improving the elimination of the smear layer, but does not increase the reaction rate.

## 5. Conclusions

Considering the results carried out by the quantitative and qualitative analysis, it is clear how it is possible to reduce the vapor lock formation with the use of PUI and how this can be almost unavoidable with the use of PPI [45]. CUI and Sonic activation can be also considered really effective methods in the vapor lock reduction. Results of this study, although characterized by low statistical power, revealed that PUI is more effective than PPI in the vapor lock reduction.

## Figures and Tables

**Table 1:** Detailed description of samples and instruments used and vapor lock formation using different irrigation techniques in each one of the three studies selected for the present systematic review.

Publication Author, Date.	sample	Final Shaping instruments	Passive Ultrasonic irrigation	Positive Pressure Irrigation	Continuous Ultrasonic Irrigation/ Sonic Activation
Castelo-Baz P et al, 2012	60 extracted teeth. closed system (20 sample for Passive ultrasonic irrigation 20 sample for Positive Pressure Irrigation, 20 for Continuous Ultrasonic Irrigation)	Protaper universal f3 (Maillefer ) of up to 30 size 0.09 taper in tips	6 Presence vapor lock	20 presence vapor lock	Continuous ultrasonic irrigation 4 presence vapor lock

Castelo-Baz P et al, 2016	60 extracted teeth closed system (20 sample for Passive ultrasonic irrigation 20 sample for Positive Pressure Irrigation, 20 for Continuous Ultrasonic Irrigation)	GTX (Maillefer) 20. 04, 20. 06, 30. 06	12 Presence vapor lock	20 presence vapor lock	Continuous ultrasonic irrigation 2 presence vapor lock

Sáinz-Pardo M et al, 2014	60 extracted teeth (30 closed system 30 Open system) 20 sample for Passive ultrasonic irrigation 20 sample for Positive Pressure Irrigation, 20 Sonic Activation)	Profile rotary files (Maillefer) of up to 30 size 0.06 taper	Open system: o presence vapor lock Closed system: 3 Presence vapor lock	closed system: 7 presence vapor lock open system: 0 presence vapor lock	Sonic activation Closed system: 6 presence vapor lock Open system: 0 presence of vapor lock

**Table 2:** Application of the Newcastle-Ottawa scale for case-control studies in order to evaluate the Risk of Bias between the three studies selected for the present systematic review.

SELECTION	Castelo-Baz P et al., 2012	Castelo-Baz P et al., 2016	Sáinz-Pardo M et al., 2014
(1) Is the case definition adequate?			
(a) yes, with independent validation*∗*	A*∗*	A*∗*	A*∗*
(b) yes, e.g. record linkage or based on self reports			
(c) no description			
(2) Representativeness of the cases	A*∗*	A*∗*	B
(a) consecutive or obviously representative series			
of cases*∗*			
(b) potential for selection biases or not stated			
(3) Selection of Controls	B	B	B
(a) community controls*∗*			
(b) hospital controls			
(c) no description			
(4) Definition of Controls	B	B	B
(a) no history of disease (endpoint)*∗*			
(b) no description of source			

COMPARABILITY			

(1) Comparability of cases and controls on the basis of the design or analysis	A*∗*	A*∗*	A*∗*
(a) study controls for vapor lock removal *∗*			
(b) study controls for any additional factor*∗*(This criteria could be modified to indicate specific control for a second important factor.)			

EXPOSURE			

(1) Ascertainment of exposure			
(a) secure record (e.g. surgical records)*∗*	E	E	E
(b) structured interview where blind to			
case/control status*∗*			
(c) interview not blinded to case/control status			
(d) written self-report or medical record only			
(e) no description			
(2) Same method of ascertainment for cases and controls	A*∗*	A*∗*	A*∗*
(a) yes*∗*			
(b) no			
(3) Non-Response rate	A*∗*	A*∗*	A*∗*
(a) same rate for both groups*∗*			
(b) non respondents described			
(c) rate different and no designation			
